# EEG-based automated evaluation of automotive sound quality using ensemble deep learning

**DOI:** 10.1038/s41598-026-58127-4

**Published:** 2026-07-24

**Authors:** Liping Xie, Min Fang, Zhien Liu, Yawei Zhu

**Affiliations:** 1 College of Mechanical Engineering & Automation, Fuzhou University, Fujian Province, Fuzhou, 350108 China; 2 Zhejiang Geely Holding Group, Hangzhou, Zhejiang 310000 China; 3 Hubei Collaborative Innovation Center for Automotive Components Technology, Wuhan University of Technology, Wuhan, 430070 China; 4 National Energy Key Laboratory for New Hydrogen-Ammonia Energy Technologies, Foshan Xianhu Laboratory, Foshan, 528200 P.R. China

**Keywords:** Automobile, Sound quality, EEG signal, Deep-ensemble learning, Auditory perception, Engineering, Mathematics and computing, Neuroscience

## Abstract

The evaluation of automotive sound quality is of considerable significance for improving driving comfort. However, existing methodologies suffer from notable limitations, including inconsistencies in subjective evaluations and weak correlations between objective metrics and auditory perception. In response to these challenges, an automated evaluation method incorporating electroencephalogram (EEG) signals and ensemble deep learning is proposed herein. Initially, EEG data is acquired from 30 subjects during exposure to 16 automobile sounds with sporty quality. Subsequently, the LSTMS-B model is incorporating Swish activation into LSTM to mitigate gradient vanishing and enhancing Bagging through optimized majority voting, achieving 90.8% accuracy with superior performance over conventional LSTM variants; Furthermore, an innovative ResNet-based regression model is developed to establish the automobile sound-EEG feature mapping, enabling the LSTMS-B model to achieve 89.75% average F1 score in sound quality classification using brain auditory representations while reducing reliance on conventional EEG paradigms. This study develops a novel sound quality evaluation paradigm through deep-ensemble learning integration, where the proposed cross-modal feature mapping method provides a transferable AI framework for interpreting human auditory perception mechanisms.

## Introduction

The evaluation of automotive sound quality is regarded as an essential methodology for optimizing in-cabin acoustic environments and developing distinctive brand sound profiles. The research results of automotive sound quality are directly related to consumers’ cognitive experience of the vehicle’s sense of luxury and technology, which has become one of the most cutting-edge research directions in the field of automotive NVH^1^. At present, the core challenges in the evaluation of automotive sound quality lie in the inherent subjectivity of auditory perception and the complexity involved in semantic interpretation^2^. Automotive sounds exhibit inherent multidimensionality in the perspective of acoustic properties, where a single auditory stimulus can simultaneously evoke multiple perceptual-semantic associations, including senses of sportiness, power, and luxury^3,4^.

Current sound quality analyses rely on subjective evaluation, which suffers from three major limitations.: First, during semantic matching, evaluators are easily influenced by individual differences—such as personal experience and cultural background. Second, linguistic descriptors (e.g., ‘thick’ or ‘sharp’) lack explicit mappings to physical acoustic parameters, hindering outcome quantification. Most critically, potential discrepancies may exist between subjective assessments and actual neural correlates of perception, making such evaluations unreliable in reflecting the brain’s cognitive processing of sound.

Neuroscientific research has demonstrated that specific types of auditory stimulation can evoke characteristic patterns in EEG responses^5,6^. For instance, s auditory perception triggers significant theta wave (4–7 Hz) energy increases in specific brain regions^7^. Quantitative analysis of indicators like power spectral density across frequency bands and functional connectivity measures enables establishing a sound-to-neural-response mapping relationship.^8,9^. Therefore, the subjective vocabulary used in conventional sound quality evaluation scales can be transformed into objective EEG-based features through the utilization of EEG signals. In this study, the combinations of EEG feature significantly correlated with automotive sounds with the quality of sporty are extracted using a neural network model, through which sound quality evaluation is achieved from a neurophysiological perspective, and new research directions for automotive sound design are provided within the neuroscience framework.

### Related works

Neural network models have been demonstrated to offer significant advantages in EEG signal processing, where deep learning architectures enable effective extraction of multi-dimensional temporal-frequency-spatial features and achieve high-precision cross-subject classification. GuoQing et al.^10^ used a backpropagation (BP) neural network to investigate the feasibility of recognizing emotion-related EEG responses induced by auditory stimulation.

Nakanishi et al.^11^ employed Fisher’s linear discriminant analysis (FLDA) to classify EEG responses evoked by three distinct audio stimulus categories using a pairwise comparison paradigm, attaining a classification accuracy of 80%. WeiLong et al.^12^ utilized a deep belief network (DBN) to develop EEG-based emotion recognition models for three affective states (positive, neutral, and negative), and the results demonstrated that using DBN-selected 12-channel EEG data as input yielded optimal classification accuracy of 86.65%. Sobhan et al.^13^ developed a hybrid CNN-LSTM architecture that directly processes raw EEG signals to extract latent features for recognizing positive, negative, and neutral emotional states. Yoon et al.^14^ developed a Bayesian classifier utilizing frequency-band-specific EEG features selected by Pearson correlation analysis, attaining > 70% accuracy in binary emotion classification. Pan et al.^15^ utilized a support vector machine (SVM) model with EEG features derived from common spatial pattern (CSP) to perform binary classification of individuals’ musical preferences. Antons et al.^16^ employed linear discriminant analysis (LDA) to assess speech quality using event-related potential (ERP) analysis, achieving a recognition accuracy of 90% across four types of speech stimuli.

In summary, existing studies have robustly demonstrated the superiority of neural network models in EEG signal recognition. And the time-frequency-space multidimensional characteristics of EEG signals can be effectively extracted through the application of deep learning techniques, which objectively quantify stimulus-induced perceptual responses (e.g., auditory processing) and establish a computationally reliable framework for investigating cognitive neural mechanisms.

In the study of auditory-evoked EEG signals, the predominant approach involves the extraction of representative EEG features, which are then directly applied to classification tasks^17^. In fact, the EEG signal acquisition stage can potentially be bypassed from the perspective of brain simulation, where features can be extracted directly, allowing human perception characteristics to be integrated with computational auditory features. In 2021, Zhang Xiao proposed an innovative ensemble-deep learning hybrid method to process EEG signals elicited by visual stimuli, establishing a mapping from visual images to EEG feature manifolds for automatic scene classification^18^. This approach overcame traditional EEG limitations by preserving perceptual features while reducing data requirements, offering a novel neural-representation-based image classification framework.

Zhang Xiao’s research has provided significant inspiration for the evaluation of sound quality^18^. It was demonstrated that the human brain exhibits analogous representation mechanisms for auditory stimuli, with EEG signals effectively reflecting neural response characteristics related to sound quality^19–21^. In this study, a novel EEG-auditory mapping approach is proposed for automotive sound quality evaluation based on the above findings. In this method, perceptual features are extracted directly from acoustic properties, and neural representations are subsequently integrated with computational acoustic analyses, thereby establishing a biologically-inspired framework for objective sound quality evaluation.

### Our contribution

In this paper, deep learning and ensemble learning techniques are combined to develop an automatic model for automotive sound quality evaluation, which demonstrates enhanced accuracy and generalization capability. The main contributions of this study are summarized as follows: (1) An integrated deep learning method is proposed to address the low classification accuracy of EEG signal algorithms for target categories, demonstrating significantly improved performance compared to conventional approaches; (2) The Swish activation function is incorporated into the traditional LSTM architecture, partially mitigating the issue of gradient vanishing. And multiple classification models are trained using the Bagging algorithm, through which the majority voting strategy is improved and the generalization capability is enhanced; (3) The proposed method is applied to automotive sound quality classification, establishing a bio-inspired mapping between human auditory perception and machine evaluation metrics. This approach enables sound quality assessment that closely replicates human auditory processing mechanisms.

The incorporation of EEG physiological signals into automotive sound quality evaluation offers three primary advantages: (1) EEG signals directly capture neural correlates of auditory perception, eliminating information loss inherent to verbal reporting; (2) quantitative EEG-acoustic feature mapping enables standardized and reproducible evaluation; and (3) neural decoding of sound quality cognition reveals fundamental mechanisms, guiding data-driven optimization in automotive acoustic design. This study provides a theoretical-technical framework for EEG-based auditory information decoding and scene reconstruction, with significant implications for auditory perception and cognition research. More importantly, the developed methodology for perceptual objectification and qualitative quantification is validated for automotive sound quality evaluation, with demonstrated extensibility to industrial acoustic design applications.

The remaining part of the paper proceeds as follows: in Sect.  2, the experimental design is described detailly. In Sect.  3, the methodology combining ensemble learning and deep learning is proposed to establish the correlation between automobile sound quality and EEG features. Section  4, the experimental results are systematically displayed and analyzed utilizing the methodology developed in Sect.  3. The summary of the full text is respectively exhibited in Sect.  5.

## Acquisition and preprocessing of EEG signals

### Acquisition of automotive acceleration stimulus sounds

30 healthy subjects are enrolled in this auditory stimulation EEG study. All subjects satisfy the following inclusion criteria: (1) normal auditory function. (2) right-handed dominance. And (3) absence of neurological disorders. The experimental protocol is funded by the National Natural Science Foundation of China (Approval No: 52175111). Written informed consent was obtained from all subjects after a full explanation of the study procedures, confirming their voluntary participation in this non-invasive EEG investigation.

**Fig. 1:**
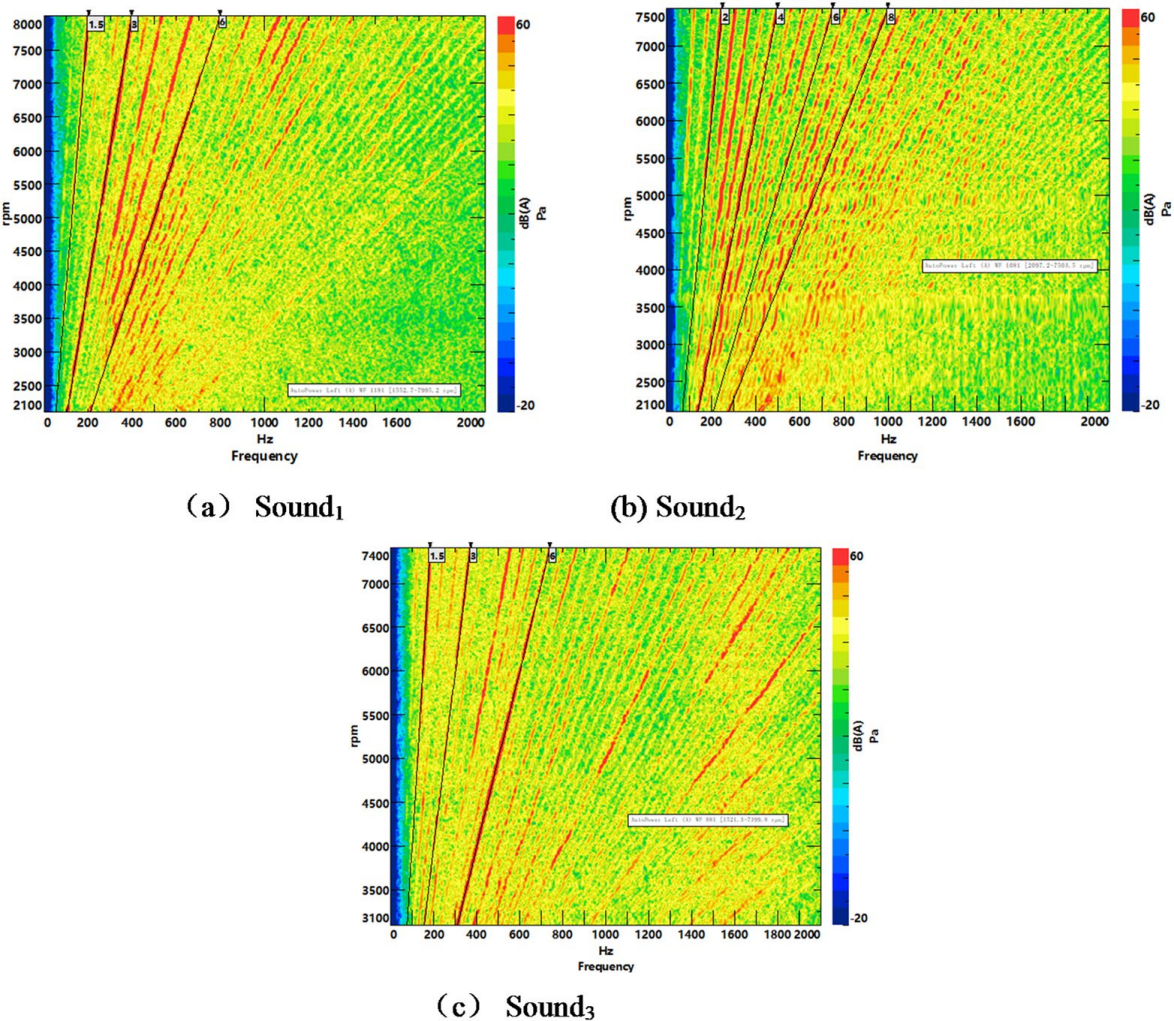
Display of three sounds among the 16-sound stimulus. (**a**) Sound1, (**b**) Sound2, (**c**) Sound3.

In this study, the in-vehicle acceleration sound signals from 16 automobiles exhibiting sporty sound quality are acquired through real vehicle acceleration tests. All recorded sounds undergo standardized preprocessing in Artemis software, including digital filtering and noise reduction. Following the above processing, 16 qualified 5-second acceleration sound signals are selected based on two objective criteria: (1) preservation of normal waveform morphology and (2) distinct order-related acoustic features. The selected sounds are systematically labeled from Sound_1_ to Sound_16_ for experimental identification. The three representative spectrograms of 16 sounds appear in Fig. 1.

A stepwise attenuation of acoustic energy is observed across the frequency range of 200–2000 Hz through the spectral analysis in Fig. 1. The highest energy concentration is identified in the low-frequency band (200–600 Hz), followed by a rapid decay in the mid-to-high frequency region (800–2000 Hz). The stimulus sounds are determined to be composed of coupled multiple sound sources, including low-frequency background noise and harmonic components. The distinctive wide-band energy distribution with smooth dynamic characteristics is found to be collectively generated by this composite multi-source architecture, representing a typical signature of sporty sound quality.

### Organization of EEG signal acquisition experiments

A 64-channel EEG system (Brain Products GmbH, Germany) is utilized for EEG data collection. Detailed instructions regarding experimental procedures and equipment operation are provided to subjects by trained researchers prior to testing. Experimental sessions are conducted in a sound-attenuated chamber, during which 16 stimulus sounds (Sound_1_-Sound_16_) are sequentially delivered to subjects via professional-grade audiometric headphones.

**Fig. 2:**
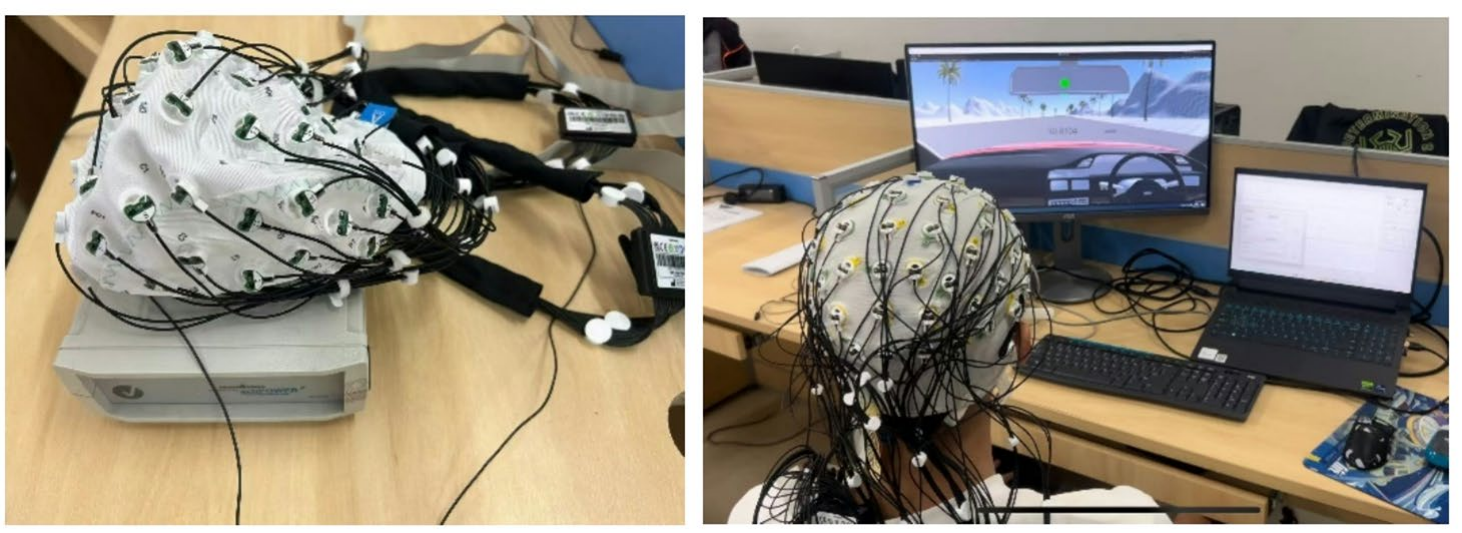
EEG acquisition equipment.

**Fig. 3:**
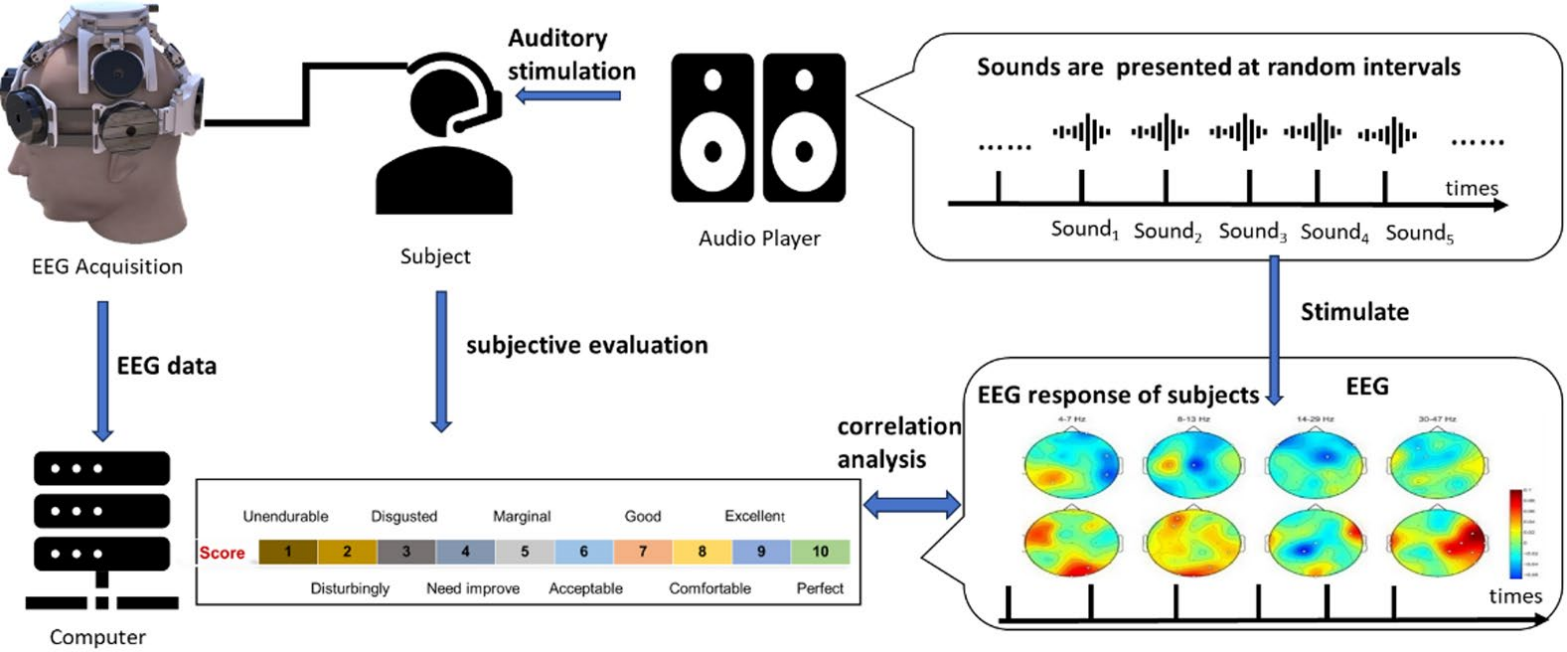
EEG acquisition flow chart.

Continuous EEG data are recorded at a sampling rate of 1000 Hz utilizing 64-channel EEG system as showed in Fig. 2. Following each sound stimulus, subjective ratings of perceived sportiness are collected using a 10-point Likert scale (1: not at all sporty and unendurable; 10: extremely sporty and perfect), with responses guided by standardized on-screen instructions. The complete experimental protocol is illustrated in Fig. 3.

**Fig. 4:**
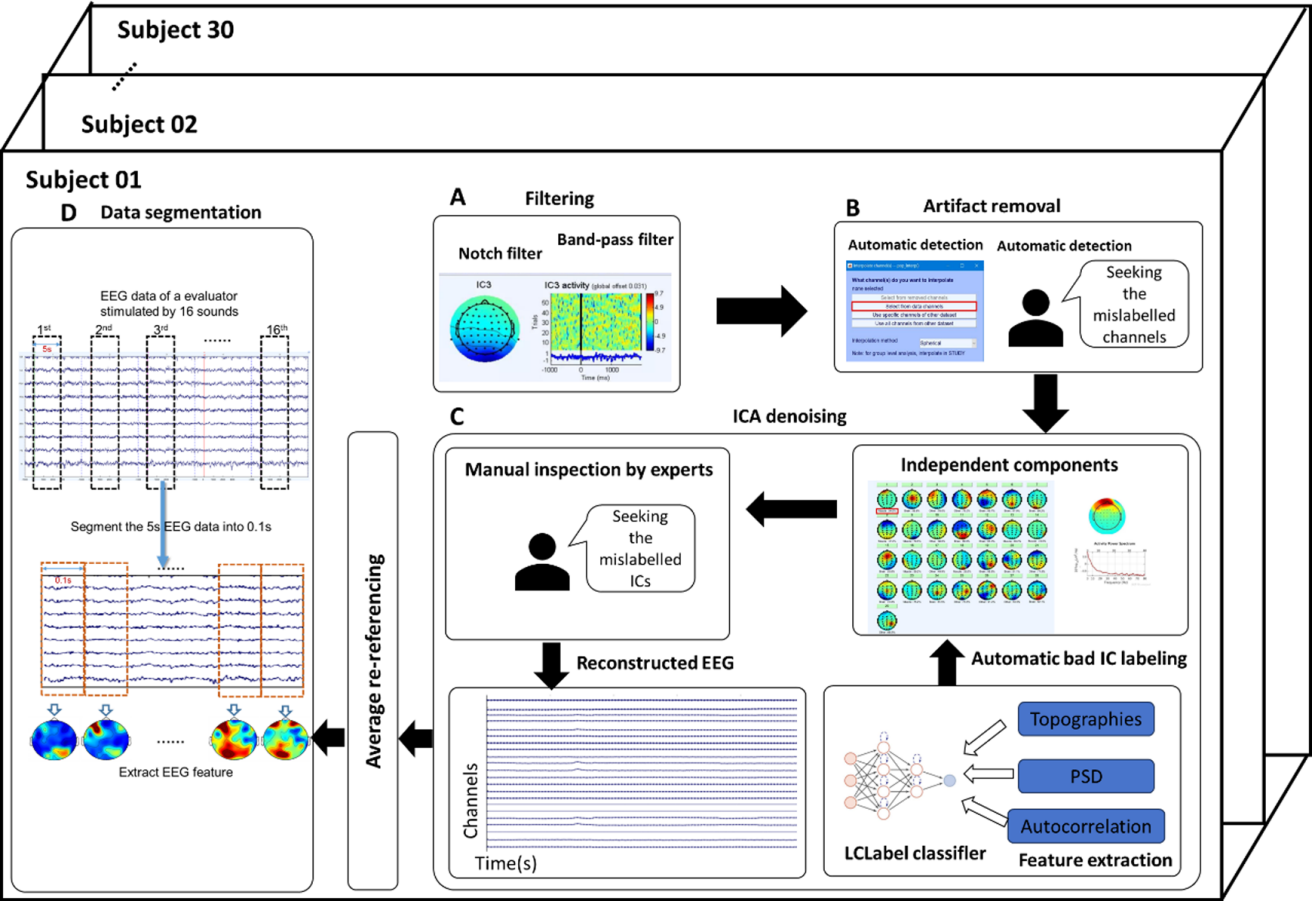
Flowchart of EEG data preprocessing.

EEG preprocessing constitutes the initial critical step in EEG data analysis. Standardized EEG preprocessing is performed using the EEGLAB toolbox, comprising the following steps: (1) Frequency-domain filtering: A 0.1–100 Hz bandpass filter is applied in conjunction with a 50 Hz notch filter to suppress line noise interference; (2) Artifact removal: Ocular artifacts (blinks and saccades) are identified and eliminated through independent component analysis (ICA); (3) Baseline correction: Zero-mean normalization is implemented to mitigate electromyographic (EMG) artifacts; (4) Re-referencing: The average potential across all scalp electrodes is adopted as the common reference to minimize positional biases; (5) Epoch segmentation: Continuous EEG recordings are partitioned into 0.1 s epochs to augment the dataset. Following this pipeline, a total of 24,000 validated EEG epochs are derived from 30 subjects (16 stimulus sounds × 5 time windows × 10s × 30 subjects). The complete workflow is illustrated in Fig. 4.

## Ensemble-deep learning hybrid methodology for automotive sound quality evaluation

**Fig. 5:**
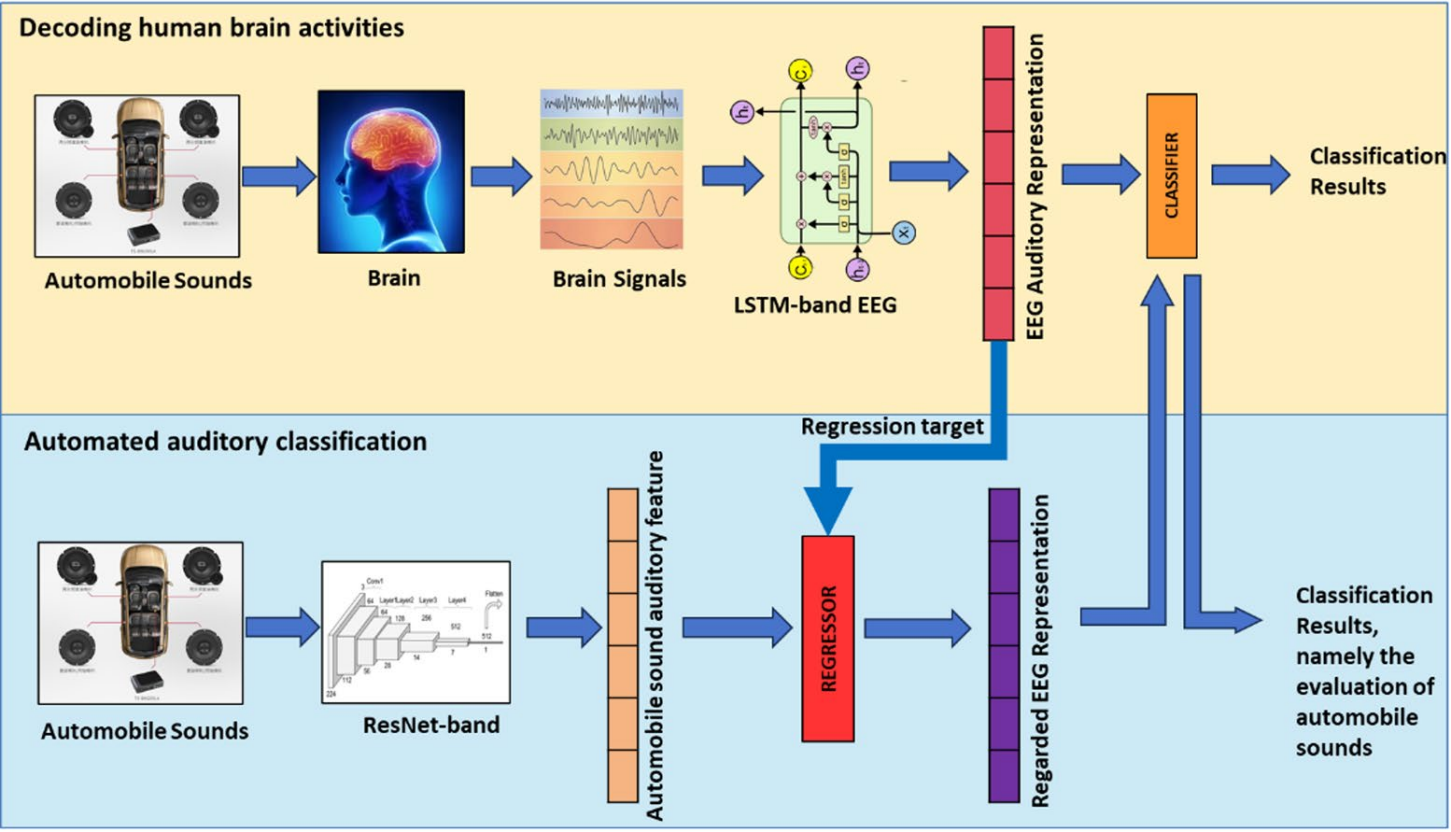
Flowchart of the proposed ensemble-deep learning hybrid methodology.

Developed as an extension of Zhang et al.‘s neural decoding architecture^18^, an integrated deep neural network architecture, based on long short-term memory (LSTM) is developed to extract low-dimensional features from high-dimensional EEG signals evoked by original automobile sounds. This framework is subsequently applied to the objective evaluation of automobile sound quality, where a ResNet-based regression model establishes a mapping between automobile sounds and low-dimensional EEG feature representations. The proposed framework is designed to achieve enhanced classification accuracy and improved cross-condition generalization capability under diverse experimental conditions. The proposed ensemble-deep learning hybrid methodology is illustrated in Fig. 5.

### EEG-based feature learning for automobile sound perception analysis

#### Enhanced LSTM architecture with swish activation function

The Long Short-Term Memory (LSTM) network—a specialized recurrent neural network utilizing unique input, forget, and output gates—is designed to simultaneously address gradient vanishing/exploding issues in long-sequence data and capture long- and short-term temporal dependencies. The capability of LSTM networks to leverage temporal dependencies and extract complex dynamical features from EEG signals has been extensively validated in neural signal processing research^22,23^.

Activation functions serve a crucial role in neural networks by introducing nonlinear transformations, which enable the networks to learn and represent complex nonlinear mapping relationships between inputs and outputs. Moreover, the efficiency of gradient propagation and model training is strongly affected by the activation function selection, since gradient vanishing/explosion issues can be alleviated, while training speed and model performance are simultaneously improved through a suitable choice. Compared to traditional activation functions like ReLU and Tanh, Swish demonstrates greater stability during optimization owing to its smoothness, whereas its non-monotonicity better captures complex feature relationships^24^.

Swish can replace traditional activation functions in the input, forget, and output gate computations of LSTM networks. The above replacement has been shown to enhance the gating mechanism’s nonlinear pattern capture in time series data while mitigating information loss during training. The continuous derivative property of Swish has been also shown to enhance gradient propagation efficiency, leading to better performance and faster convergence in EEG time-series analysis. Its mathematical formulation is given in Eq. (1):

1 $$Swish(x) = x\sigma (\beta x)$$

**Fig. 6:**
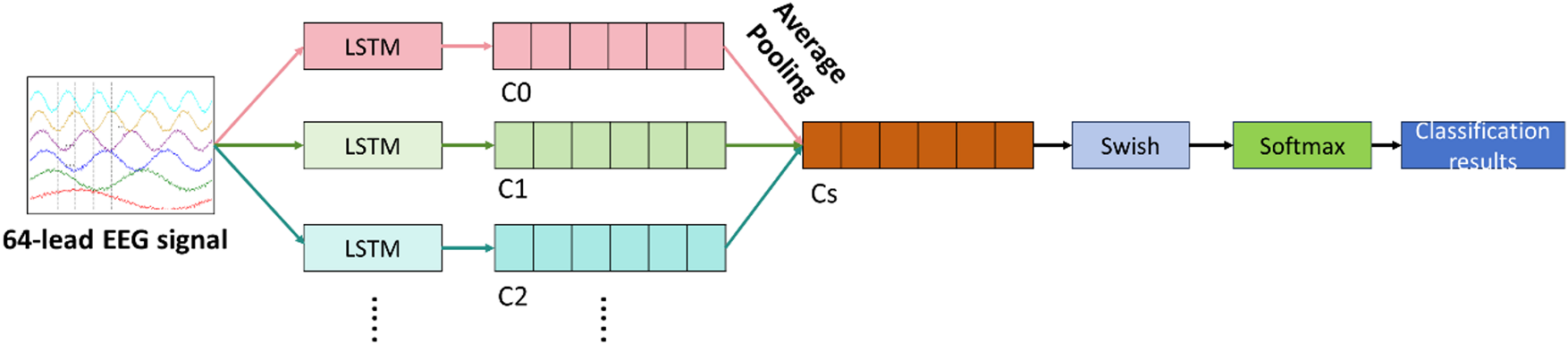
The classification of Automotive sound quality based LSTMS model.

In this formulation, σ(x) denotes the Sigmoid function. Swish can dynamically transition between Sigmoid and ReLU by adjusting the parameter *β*, thereby allowing greater flexibility in adapting to the requirements of different tasks. The Swish function is smooth and continuously differentiable, which contributes to improved stability during backpropagation. An LSTMS model integrating LSTM with Swish activation is proposed in this study, capitalizing on Swish’s advantages, as illustrated in Fig. 6.

The LSTM network is designed with an input size of 64 to process the 1 × 64 dimensional EEG signal segments at every time step. All hidden layer outputs of the LSTM are processed by an average pooling layer, and the resulting intermediate vector is denoted as Cs. The vector Cs is then passed through the Swish activation function and subsequently classified into 10 categories using a Softmax layer, corresponding to sound quality evaluation scores ranging from 1 to 10. The output of each LSTM model is regarded as the auditory feature vector of the EEG signal, which serves as the input to the corresponding Softmax classifier.

#### An improved bagging algorithm

Ensemble learning is a technique used to enhance overall performance by combining the prediction results of multiple models, wherein multiple weak learners are integrated into a single strong learner. The Bagging and Boosting approaches are predominantly included among common ensemble learning methods. The core principle of Bagging is to (1) generate multiple distinct training subsets via random sampling with replacement, (2) train independent base models on each subset, and (3) aggregate all predictions to reduce variance and improve generalization. The final prediction based on the Bagging principle can be expressed as:

Classification tasks:

2 $$\hat{y} = mode(\hat{y}_{1} ,\hat{y}_{2} ,...,\hat{y}_{m} )$$

Where *mode* represents the majority vote and y^_m_ is the prediction result of the *i*^th^ base model.

Regression tasks:

3 $$\hat{y} = \frac{1}{m}\sum\nolimits_{{i = 1}}^{m} {\hat{y}_{i} }$$

The final prediction is generated through averaging of all base model outputs. The combination of bootstrap sampling and prediction aggregation enables Bagging to significantly reduce the variance error inherent in individual models.

**Fig. 7:**
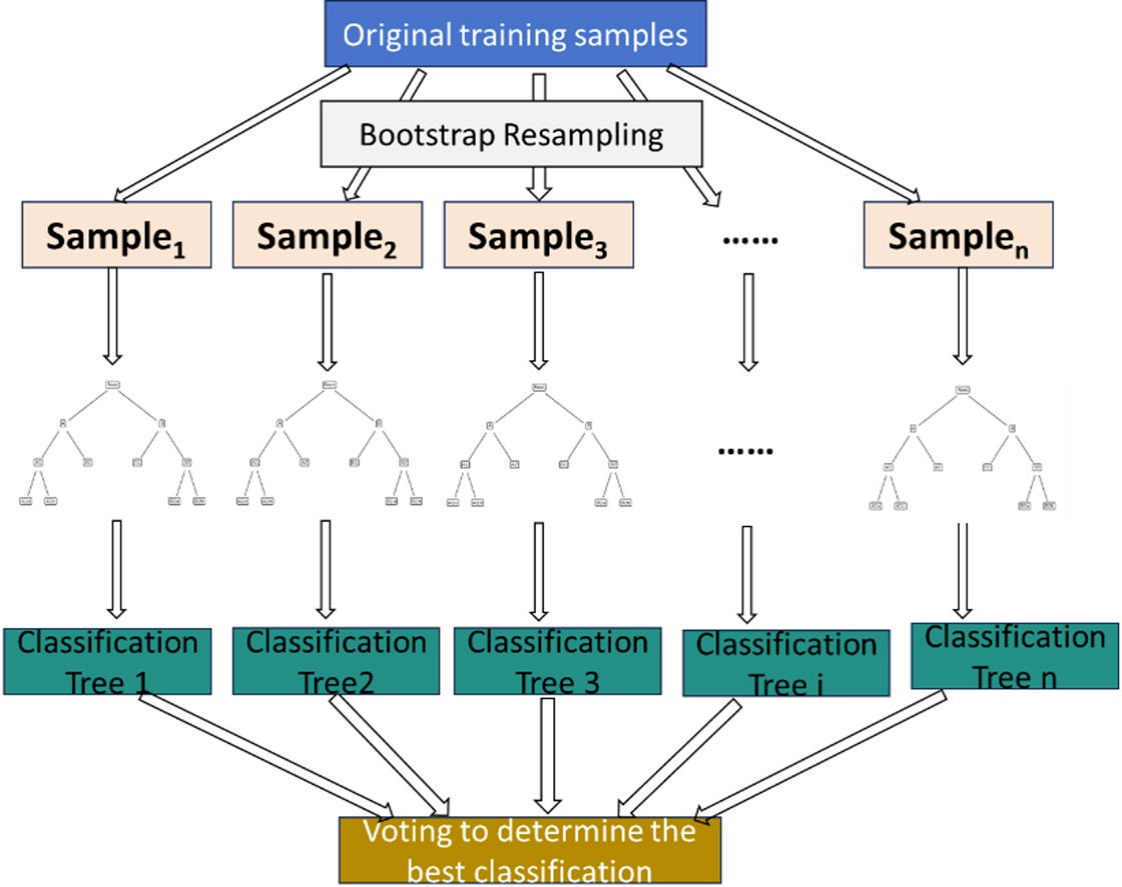
Schematic diagram of the Bagging algorithm.

Bootstrapping is a statistical method relying on random sampling with replacement, widely used to assess model stability and build ensemble models. As the theoretical foundation of Bagging, this method operates by repeatedly resampling the original dataset to generate multiple data subsets, thus approximating the population distribution characteristics. The data excluded from bootstrap samples are termed Out-of-Bag (OOB) data, which serve as an intrinsic validation set for performance evaluation. In the proposed method, the generalization ability of each base classifier is estimated through OOB error analysis. The schematic diagram of the Bagging algorithm is showed in Fig. 7.

In conventional bagging, the final prediction is obtained through majority voting, where all base classifiers are assigned equal weights. However, this approach fails to account for performance variations among individual classifiers. To mitigate this issue, conventional enhancement methods assign distinct weights to each base classifier, where higher weights are allocated to classifiers exhibiting superior performance^25,26^. An improved voting strategy based on Out-of-Bag (OOB) data is proposed, in which heterogeneous classification capabilities of base classifiers across different categories are considered. Specifically, category-specific weights for each classifier are determined by utilizing the F1-scores derived from OOB data.

Firstly, the reliability coefficient *C* of each base classifier is calculated for different data categories using the OOB data. The calculation formula is presented as follows:

4 $$C_{{{\mathrm{in}}}} = \frac{{OOB\_F_{{in}} }}{{\sum\nolimits_{{n = 1}}^{N} {OOB\_F_{{in}} } }}$$

In the formula, *i* denotes different categories. For the dataset employed in this study, *i* ranges from 1 to 10. The reliability coefficient of the *n*^th^ base classifier for the *i*^th^ category is denoted as C_*in*_, which is calculated based on the F1-score (OOB_F_in_) obtained from the Out-of-Bag (OOB) data of the *n*^th^ classifier for the *i*^th^ category, where *N* represents the total number of base classifiers.

The coefficient C_*in*_ exhibits the following two characteristics: (1) The coefficient value increases with improved classifier performance on the dataset; (2) The normalization constraint $\sum\nolimits_{{n = 1}}^{N} {C_{{{\mathrm{in}}}} } = 1$ is imposed, ensuring that the variance among reliability coefficients remains bounded for all base classifiers within the same category.

In the proposed method, the reliability coefficient *C* is employed as the category-specific weight for each base classifier, thereby mitigating excessive reliance on high-performing classifiers while preserving the core principle of collective decision-making in the Bagging framework.

#### A LSTMS-B model integrating LSTMS enhancement and adaptive bagging optimization

**Fig. 8:**
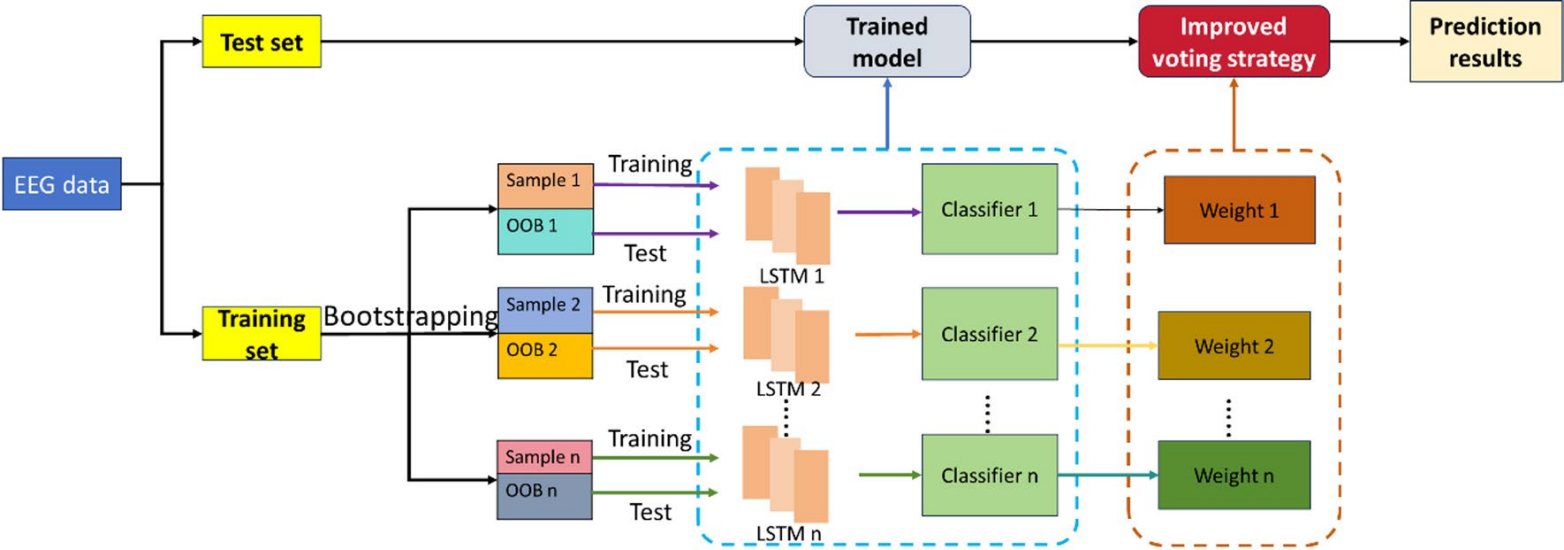
Flowchart of the LSTMS-B algorithm framework.

The LSTMS-B model for human brain activity decoding is proposed, which is developed based on the on the research framework of Zhang et al.^18^. The data flow architecture of the proposed model is illustrated in Fig. 8.

The LSTMS-B model combines the idea of ensemble learning with the deep learning model. The conventional Bagging algorithm framework is adopted, in which an LSTM model incorporating the Swish activation function is implemented as the base classifier for human brain activity decoding. Subsequently, the Out-of-Bag (OOB) data are leveraged to calculate category-specific weights for individual classifiers, and an enhanced voting strategy is formulated specifically for vision-related EEG signal decoding and classification^27^. The detailed algorithmic procedure is presented as follows:

1. Data Partitioning: The EEG dataset is partitioned into training and test sets based on sound stimuli, with each EEG sample labeled according to its corresponding sound quality category.
2. Bootstrap Sampling: T bootstrap samples are generated through random sampling of the training set.
3. Classifier Training: For each of the T training samples, an LSTMS model is employed to train a Softmax classifier, thereby establishing T weak classifiers.
4. OOB Data Collection: The Out-of-Bag (OOB) data corresponding to each bootstrap sample are collected.
5. Reliability Calculation: The reliability coefficient C for each weak classifier across different categories is computed using both the trained classifiers from Step 3 and the OOB data from Step 4.
6. Iterative Training: Steps 3 through 5 are repeated until all weak classifiers are trained and their corresponding weights are obtained.
7. Ensemble Prediction: All weak classifiers are integrated with their respective weight distributions, and the test set categories are predicted using the improved voting strategy. The output from each LSTMS model, serving as input to its corresponding Softmax classifier, is utilized as the EEG feature vector for accuracy calculation.

### Regression modeling of automotive sound-EEG feature relationships

The both LSTMS network and its paired Softmax classifier are successfully trained through the introduction in Chap. 3.1. The output generated by each LSTMS model, where this output corresponds to the input of each Softmax classifier, serves as the EEG-derived feature vector encoding neural responses to automotive sound stimuli.

An alternative approach is introduced to generalize the LSTMS-B-learned EEG features to wider automotive sound stimuli: EEG signal acquisition is bypassed by directly predicting auditory-evoked neural patterns from acoustic features. Given that the learned EEG features can effectively represent the evoking auditory stimuli, a ResNet-based approach is employed to extract approximate EEG features directly from automotive sound inputs, constituting a regression process.

**Fig. 9:**
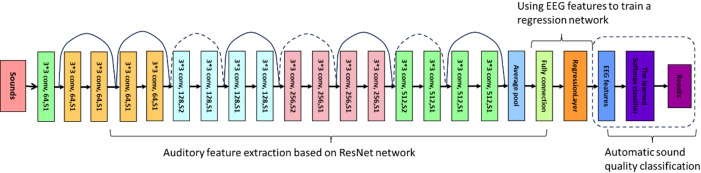
ResNet-based regression model.

The ResNet architecture has been widely adopted for image detection, segmentation, and recognition tasks owing to its simplicity and practical effectiveness^28^. In this framework, residual blocks are systematically interleaved across network depths, where skip connections counteract gradient vanishing and representational saturation. Consequently, the proposed regression analysis methodology comprises a ResNet model integrated with a regression network. The ResNet-based regression architecture is illustrated in Fig. 9.

The training pipeline consists of two stages: Initially, acoustic features associated with automotive sounds are extracted using the pre-trained ResNet-18 model. Subsequently, these acoustic features are mapped to EEG features through regression analysis. And K-Nearest Neighbor (KNN) is employed to map auditory features to EEG features. The regression layer is configured with neuron quantities matching the dimensionality of the EEG features extracted by the LSTMS-B model.

A trained regression model capable of predicting EEG features from arbitrary automotive sounds is obtained via the proposed methodology. These features are subsequently classified using the Softmax classifier trained as introduced in Sect.  3.1, enabling automobile sound quality evaluation based on EEG signal sources.

## Method validation and experimental results

This Section is intended to analyze and validate the innovative research concept of integrating EEG signals into the evaluation of automobile sound quality as proposed in this study.

Firstly, the analysis of EEG feature effects related to automobile sound quality is carried out based on the LSTMS-B integrated deep learning method proposed in Sect.  3.1; Subsequently, the performance of the EEG feature regression model based on the ResNet architecture constructed in Sect.  3.2 is analyzed, and the feasibility and effectiveness of the EEG-driven sound quality evaluation approach is verified. The model is trained on a workstation with an Intel i9-13900 K processor (3.7 GHz) and an NVIDIA RTX 3080 graphics card (10 GB GDDR6X).

### Learning EEG acoustic features using the LSTMS-B model

To validate the generalization ability of the proposed LSTMS-B model in extracting EEG features associated with automotive sound quality, a comparative experiment is designed in this section, in which the analysis is conducted from the perspectives of activation function optimization and integration strategy enhancement.

First, to evaluate the advantages of the Swish activation function within the LSTM structure, three model variants are established for comparison: (1) the original LSTM model, (2) the LSTM model employing the ReLU activation function (termed LSTM+ReLU), and (3) the LSTMS proposed in this study; Secondly, to validate the effectiveness of the improved Bagging method, the traditional simple majority voting strategy is introduced to construct the comparison model LSTMS-MV (i.e., integration based on the “majority rule” principle), while the LSTMS structure remained unmodified; Finally, 5 model variants—LSTM, LSTM+ReLU, LSTMS, LSTMS-MV, and the proposed LSTMS-B—are implemented for experimental comparison to comprehensively evaluate model performance and enhancement effects.

The above 5 model variants are trained using the back-propagation algorithm, and the parameter gradients are calculated via the mini-batch gradient descent method to optimize model performance. The EEG dataset consist of 24,000 samples of automotive sound-related neural responses, which were partitioned into training and test sets at an 8:2 ratio (19,200 and 4,800 samples, respectively). In addition, to enhance the generalization and robustness of the model, 10 random sampling operations with replacement are performed on the training set to construct sub-training sets for the ensemble learning model. The average classification Accuracy, Recall rate, and F1 score of the five models are calculated, and the results are presented in Table 1.

**Table 1:** Comparative analysis of model performance (%).

Model	Accuracy	Recall rate	F1 score
LSTM	0.782	0.770	0.776
LSTM+ReLU	0.812	0.800	0.806
LSTMS	0.861	0.852	0.856
LSTMS_MV	0.886	0.874	0.880
LSTMS-B	0.912	0.905	0.908

As evidenced by the experimental results in Table 1, statistically significant performance variations are observed across the compared models. Among the compared models, the proposed LSTMS-B demonstrated a 2.80% improvement in F1-score (from 0.880 to 0.908) over the LSTMS-MV using conventional majority voting, which indicates that the OOB-optimized integration strategy offers superior capability for model performance improvement. In terms of model structure, the Swish-activated LSTM achieved an 8% higher F1-score (0.856 vs. 0.776) compared to the baseline LSTM, conclusively demonstrating the enhanced nonlinear feature representation capability provided by this activation function.

**Fig. 10:**
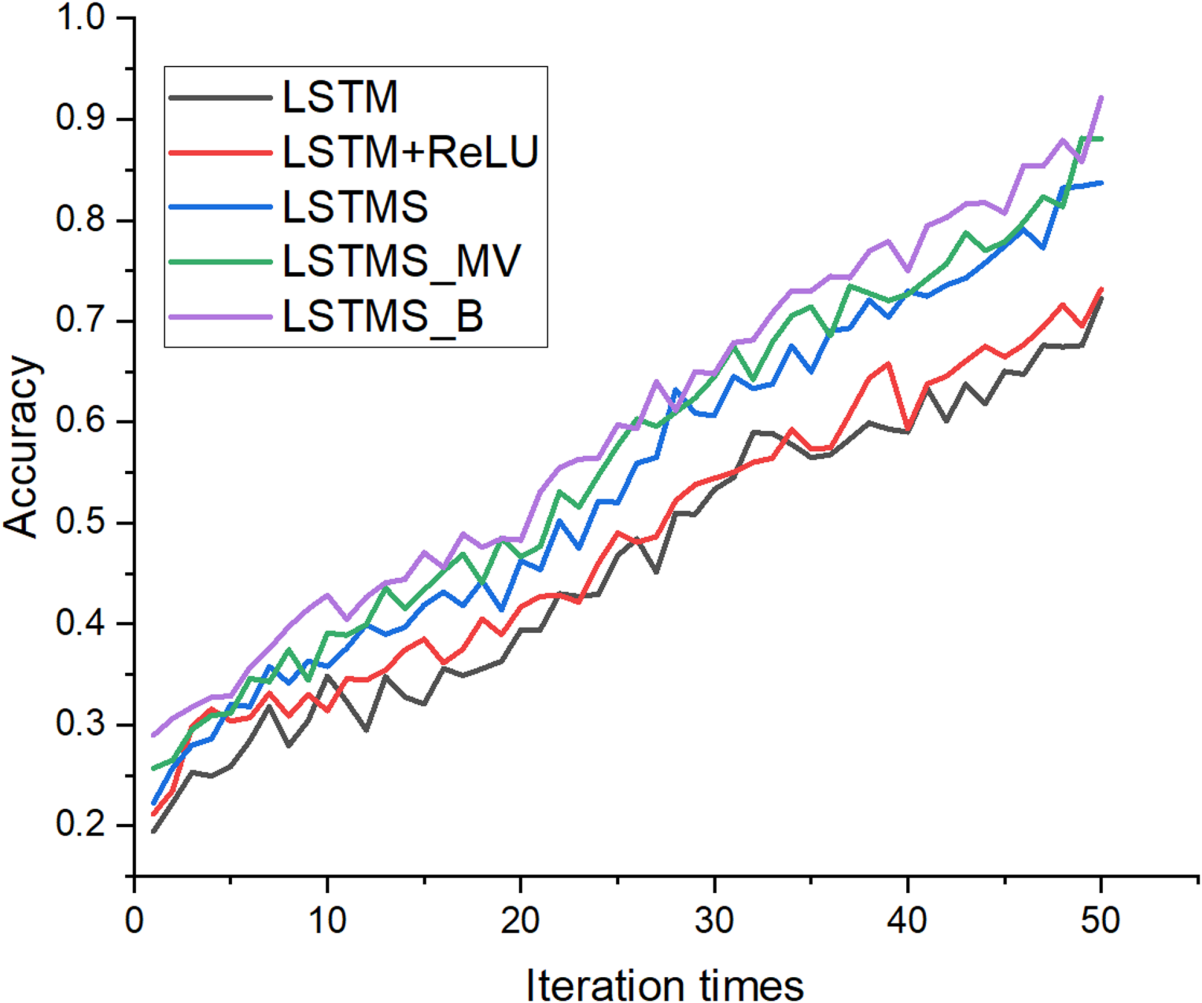
Trend of model classification accuracy during training process.

Overall, the best results in the three key evaluation metrics (Accuracy, Recall rate, and F1-score) are achieved by the LSTMS-B model, thereby confirming the superiority and robustness of the deep learning fusion combined with the improved ensemble learning strategy proposed in this study for EEG-based automotive sound evaluation. The superior performance of the proposed LSTMS-B model is further demonstrated in Fig. 10.

The experimental results demonstrate that classification accuracy of all five models exhibits a consistent increasing trend with training epochs, eventually reaching stabilization during later training phases. Specifically, the traditional LSTM model achieves relatively weak performance, with an accuracy of approximately 72% at the 50th training epoch, and the ReLU-activated LSTM achieves a marginally higher accuracy of 74%, though the improvement is not significant. In contrast, the Swish-activated LSTM manifests a significant performance advantage (85.5% accuracy), outperforming the baseline LSTM by 13.5%, which fully demonstrates the superior performance of the Swish function in enhancing gradient propagation and the model’s nonlinear representation capability.

Although the LSTMS model achieves competitive performance, it underperforms compared to the ensemble models. Specifically, an accuracy of 88% is obtained by the majority-voting LSTMS-MV, compared to 91% achieved by the LSTMS-B model implementing the OOB optimization strategy introduced in this study.

The above results demonstrate that the improved Bagging strategy boosts performance by 5.5% over the baseline LSTM, with an additional 3% accuracy gain compared to majority voting (88%→91%), confirming the efficacy of our ensemble method for EEG classification. The result not only indicates that the improved Bagging strategy enhances model performance by 5.5%, but more importantly, it provides an additional 3% gain over the traditional voting method, thereby strongly confirming the significant advantages of the optimized multi-classifier integration scheme in EEG signal classification tasks.

### Automotive sound quality prediction from EEG signals based on ResNet-regression model

A ResNet-based regression module is designed to establish mapping relationships between the acoustic features of automobile sounds and the EEG features extracted by the LSTMS-B model. The LSTMS-B model first extracts key EEG features, based on which the sound quality category is determined. Each automotive sound stimulus is transformed by the LSTMS model into a 64-dimension EEG feature vector, and the ResNet regression network is then designed to map acoustic features (input) to these EEG features (target output), preserving the 64-dimensional vector space. The distribution of the regression output is expected to closely approximate the EEG feature vector extracted by the LSTMS-B model, thereby enabling an effective mapping from automotive sound to EEG-based perceptual features.

**Fig. 11:**
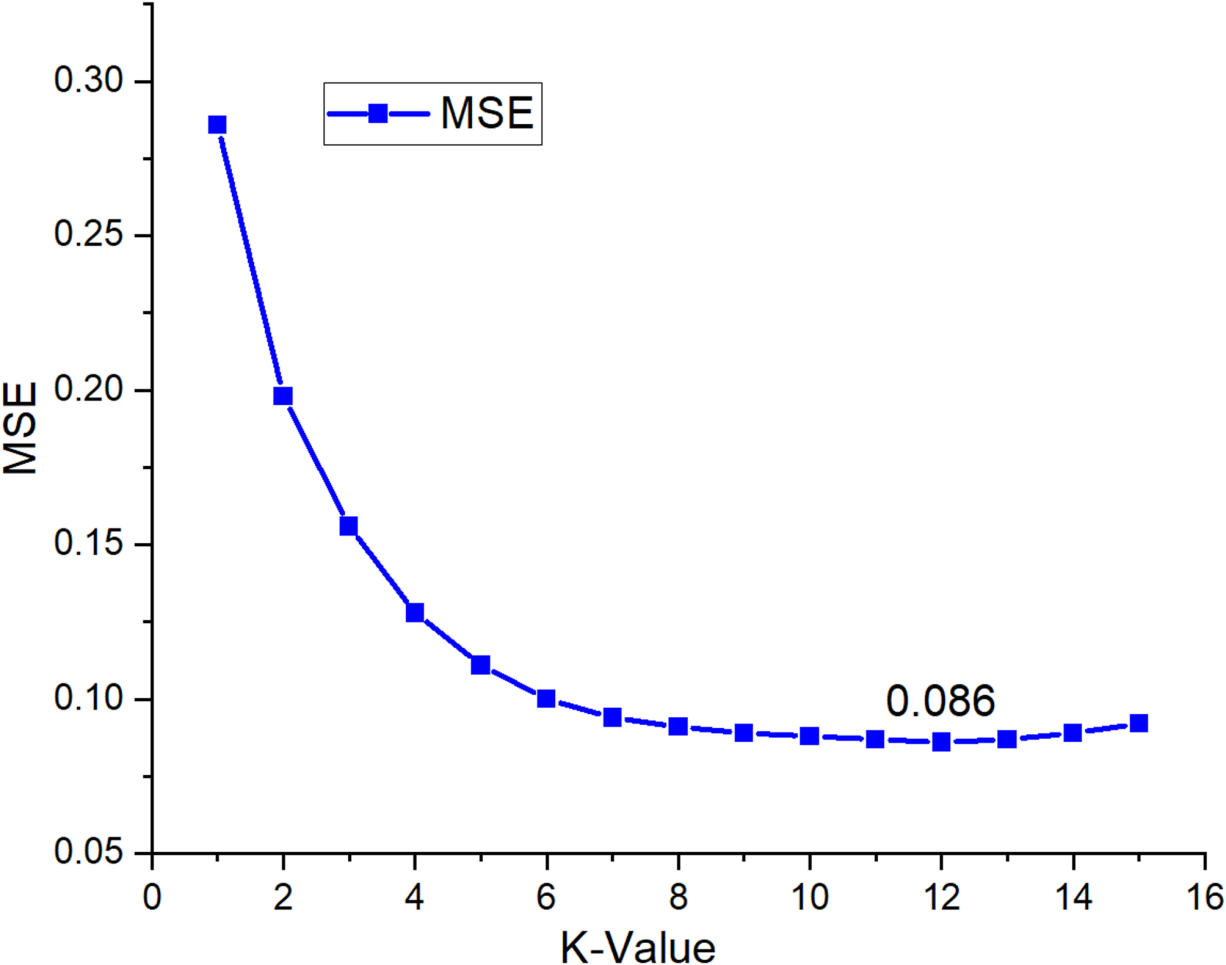
The impact of k-value (1–15) on k-NN regression performance.

To evaluate the performance of the regression model constructed in this study, the sounds used to induce EEG signals are divided into training and test sets using the same method as described in Sect. 4.1. Additionally, Mean Square Error (MSE) is adopted in this study as the evaluation metric for assessing the performance of the regression model. Experimental results demonstrate that ResNet combined with k-NN regression achieves the lowest MSE loss^18^.The impact of k-value (1–15) on k-NN regression performance is verified through MSE calculations, as presented in Fig. 11.

A clear inverse relationship between k-value and MSE is observed in Fig. 11 dictating that generalization performance is improved with higher neighbor counts. The lowest MSE of 0.086 is achieved at k = 12, followed by slight fluctuations and increases, suggesting that excessively large K values may introduce redundant information and reduce regression accuracy. These results highlight the criticality of optimal k-value selection for k-NN regression enhancement, while demonstrating the efficacy of ResNet-based feature extraction combined with k-NN.

The simulated EEG features generated by the ResNet-KNN model are used as input and fed into the pre-trained Softmax classifier from LSTMS-B model, thereby enabling the final evaluation of automobile sound quality. A performance comparison is presented in Table 2 between the LSTMS-B integrated ResNet-k-NN model and the reference SVM and CNN-LSTM models from previous research^29^.

**Table 2:** A performance comparison between the proposed model and the previous research.

Model	Sensitivity	Precision	Accuracy	Macro-F1
SVM^29^	71.78%	81.77%	73.50%	76.00%
CNN+LSTM ^29^	84.58%	87.08%	85.50%	85.40%
Our Model	90.20%	89.30%	89.70%	89.75%

Overall, the proposed model demonstrates superior performance compared to the comparison models in terms of sensitivity (90.20%), precision (89.30%), overall accuracy (89.70%), and Macro-F1 score (89.75%), indicating enhanced recognition capability and classification stability. Compared with the SVM model, the overall accuracy of the proposed model is increased by 16.2%, and the Macro-F1 score is improved by 13.75%, indicating the limitations of traditional machine learning methods in processing EEG data. Compared to the CNN+LSTM deep learning model, the proposed method achieves further improvement in classification performance, thereby verifying the effectiveness and robustness of the model in extracting EEG feature. The above results demonstrate the higher practical value and application potential of the proposed approach in target recognition tasks, supporting the validity of classifying sound quality based on EEG signal sources.

In future research, the research methods presented in this paper can be further extended to the field of automotive sound design. For instance, acoustic shortcomings are identified through EEG analysis- such as the alpha amplitude reduction of EEG feature, guiding focused improvements in the 200–500 Hz band when the sportiness ratings of sound quality are suboptimal.

## Discussion

In this study, neural network technology from the field of computational science is utilized to process and analyze EEG signals evoked by the stimuli of automobile sounds. The correlation between EEG responses and acoustic features of automobile sounds is investigated, with the aim of establishing a mapping between human auditory perception and sound characteristics. Several key research questions are addressed to advance understanding in this domain.

Brain-computer interface (BCI) technology enables real-time capture of electrophysiological activity in the cerebral cortex, allowing objective decoding of neural response characteristics during auditory cognition, which provides objective physiological evaluation metrics for sound quality research^30–32^.For instance, the amplitudes and latencies of auditory evoked potentials (e.g., N1 and P2 components) are analyzed to quantify the degree of cortical activation induced by different frequency sounds. Alternatively, by examining event-related synchronization phenomena, the correlation patterns between vehicle sound comfort levels and γ-band oscillations can be revealed^33^. This neuroscientific approach not only overcomes the subjective limitations of traditional evaluation methods but also elucidates the formation mechanisms of sound quality preferences from a neural cognitive perspective^34^. These findings establish the foundation for a multidimensional evaluation model integrating acoustic parameters, neural responses, and subjective experiences.

Current research on automobile sound quality classification using EEG signals is confronted with two primary challenges. Firstly, effective extraction of stimulus-relevant features from complex EEG signals is difficult to achieve through conventional methods^17^. Secondly, the generalization capability of existing machine learning models is significantly limited by individual variability and noise interference^35^. In response to these challenges, an innovative LSTMS-B analytical method is proposed in this study. Experimental results demonstrate that the LSTMS-B model achieves a classification accuracy of 90.8% for EEG signals evoked by vehicle sounds, showing significant improvements over conventional LSTMs and LSTM+ReLU architectures. The model is shown to adaptively capture both temporal common features and subject-specific characteristics in EEG signals through its hierarchical feature extraction and weight-sharing mechanisms, effectively overcoming the limitations associated with manual feature extraction. Compared with existing approaches^35^, this method not only validates the efficacy of deep learning in EEG feature extraction, but also provides a robust solution with enhanced generalization capability for cross-subject sound quality classification scenarios.

On the other hand, the traditional evaluation of automobile sound quality relies on subjective experiments or manual design of acoustic features, which has the drawbacks of high cost and poor scalability. In this study, an innovative mapping between EEG features and acoustic characteristics is proposed through a ResNet regression model, establishing an automated evaluation framework based on human auditory neural representations. The framework employs EEG features extracted by the LSTMS-B model as intermediate representations, with a nonlinear relationship between vehicle acoustic parameters and neural responses being established through the ResNet architecture. This proposed approach effectively circumvents the need for repetitive large-scale EEG data collection in subsequent studies. Experimental results demonstrate that an F1 score of 89.75% is achieved for sound quality classification when ResNet-predicted EEG features are input into the LSTMS-B model, closely approximating the classification performance obtained with actual EEG data. Compared with existing approaches, the principal innovation of this study is demonstrated through the integration of neuroscientific mechanisms into sound quality evaluation modeling^18,35^.

In summary, automatic classification and evaluation of automobile sound quality are successfully achieved through the novel integration of the LSTMS-B model with ResNet regression, utilizing EEG signals. This approach is demonstrated to provide a new methodological perspective for the application of neural signals in automotive acoustic quality assessment. These findings not only confirm the feasibility of electroencephalogram-based sound quality identification, but also establish a foundation for intelligent vehicle acoustic design and the development of personalized in-vehicle sound systems.

However, as an initial investigation into the neural correlates of automobile sound quality perception, several limitations are acknowledged in this study: Firstly, the diversity of sound quality categories and acoustic samples should be increased, with expansion of the test population size and optimization of demographic balance across age and occupational groups. Secondly, more ecologically valid experimental scenarios could be constructed through driving simulator platforms to examine environment-dependent perceptual differences. These enhancements are expected to facilitate broader applications of EEG signal analysis in automotive acoustic engineering.

## Conclusion

This study systematically investigates the integration of ensemble learning and deep learning for processing EEG responses to automotive sounds.

EEG data are acquired from 30 subjects under the controlled auditory stimulation conditions, employing 16 automotive sound stimuli with sporty quality. Subsequently, the LSTMS-B framework is developed for automated classification and evaluation of automotive sound quality using EEG signals. To address gradient vanishing issues, the Swish activation function is incorporated into the LSTM architecture, and the model’s generalization capability is enhanced through simultaneous optimization of the LSTM network and Bagging algorithm, including modification of the conventional majority voting strategy. Experimental results demonstrate that the proposed LSTMS-B method achieved 90.8% average classification accuracy for EEG patterns evoked by automotive sounds, significantly outperforming conventional LSTM, LSTM+ReLU, LSTMS, and LSTMS-MV model; Furthermore, a novel ResNet-based regression architecture is developed to integrate the LSTMS-B model with acoustic features of automotive sounds. This cross-modal framework enables the system to emulate human auditory processing patterns, thereby achieving brain-inspired sound quality assessmen. The experimental results demonstrate that using ResNet-predicted auditory EEG features as input to the LSTM-SB model achieves an average F1 score of 89.75%, confirming the efficacy of neural activity-derived features for sound quality classification. This study establishes a methodological framework for AI-based investigation of human auditory perception mechanisms.

## Data Availability

The datasets used and/or analyzed during the current study are available from the corresponding author on reasonable request.
